# Neuroprotection mediated by ST266 requires full complement of proteins secreted by amnion-derived multipotent progenitor cells

**DOI:** 10.1371/journal.pone.0243862

**Published:** 2021-01-06

**Authors:** Keirnan Willett, Reas S. Khan, Kimberly Dine, Howard Wessel, Ziv Z. Kirshner, Jodie L. Sauer, Ashley Ellis, Larry R. Brown, Kenneth S. Shindler

**Affiliations:** 1 Department of Ophthalmology, Scheie Eye Institute, University of Pennsylvania, Philadelphia, Pennsylvania, United States of America; 2 Noveome Biotherapeutics, Inc., Pittsburgh, Pennsylvania, United States of America; Universidade Federal do ABC, BRAZIL

## Abstract

ST266 is the biological secretome of cultured Amnion-derived Multipotent Progenitor cells containing multiple growth factors and cytokines. While intranasally-administered ST266 improves the phenotype in experimental optic neuritis, specific ST266 components mediating these effects are not known. We compared the effects of ST266 with and without removal of large molecular weight proteins both in vitro and in the multiple sclerosis model experimental autoimmune encephalomyelitis (EAE) in C57BL/6J mice. Mice were treated daily with intranasal vehicle, ST266 or lower molecular weight fraction of ST266. Retinal ganglion cells were counted in isolated retinas, and optic nerves were assessed for inflammation and demyelination. ST266 treatment significantly improved retinal ganglion cell survival and reduced optic nerve demyelination in EAE mice. The lower molecular weight ST266 fraction significantly improved optic nerve demyelination, but only showed a trend towards improved retinal ganglion cell survival. ST266 fractions below 50kDa increased Schwann cell proliferation in vitro, but were less effective than non-fractionated ST266. Demyelination attenuation was partially associated with the lower molecular weight ST266 fraction, but removal of higher molecular weight biomolecules from ST266 diminishes its neuroprotective effects, suggesting at least some high molecular weight proteins play a role in ST266-mediated neuroprotection.

## Introduction

Acute demyelinating optic neuritis is the most common presenting symptom of multiple sclerosis (MS), a debilitating demyelinating disease of the central nervous system and leading cause of neurologic disability in young adults [1]. Corticosteroids—a commonly used therapy for optic neuritis—can potentially accelerate visual recovery, but does not meaningfully alter the final visual outcome, with approximately 60% of patients developing some permanent visual deficits, including 40% of affected eyes which do not return to baseline visual acuity [2]. Novel interventions are needed to improve morbidity in these patients.

Recent work has shown therapeutic potential for ST266 (formerly ACCS, Amnion-derived Cellular Cytokine Solution) [3–7], a biologic therapy containing secreted products from human placenta Amnion-derived Multipotent Progenitor (AMP) cells. ST266 contains hundreds of biomolecules, including numerous growth factors and cytokines and other common components of known anti-inflammatory and anti-apoptotic pathways [3].

Intranasally-delivered ST266 protects against vision loss, preserves retinal ganglion cells (RGCs), and decreases inflammation and demyelination of the optic nerve in the experimental autoimmune encephalomyelitis (EAE) mouse model of MS [4, 6]. These effects are selective for EAE optic neuritis, with the highest concentrations of ST266 proteins accumulating preferentially in the eye and optic nerve, with no effects observed in suppressing EAE lesions in the spinal cords of the same mice [4]. Daily intranasally-delivered ST266 also exerts similar neuroprotective effects in a model of traumatic optic neuropathy [6].

The mechanism by which ST266 decreases demyelination and promotes neuronal survival is not fully understood but is likely multifactorial and is being actively investigated. While the total ST266 secreted protein concentration from the AMP cells is in the microgram per ml range, individual components are only present in pg to ng per ml quantities [7]. Liquid chromatography—mass spectrometry studies indicate that there are hundreds of cytokines and growth factors in ST266. Therefore, it is difficult to specifically identify which individual factors are necessary to promote neuroprotection. Evaluation of ST266 molecular weight fractions might facilitate further understanding of the relative role of some of the factors found in this biologic therapy. One consideration in the formulation of ST266 is the requirement for large molecular weight proteins. AMP cells that secrete ST266 are cultured in proprietary STM100 growth media containing human serum albumin (66.5 kDa) which acts as a stabilizer that prevents protein aggregation and adsorption of cytokines and growth factors to plastic containers and pipettors [8]. While albumin is not expected to play a significant role in neuroprotection, the potential role of other high molecular weight molecules in ST266-mediated neuroprotective effects is unknown.

To examine the relative role of large and presumably inert proteins such as albumin as well as higher molecular weight growth factors in the neuroprotective and myelin-protective effects mediated by ST266 in optic neuritis, we generated a lower molecular weight fraction of ST266 in which proteins larger than 50 kDa were excluded. Effects of this less than 50 kDa ST266 fraction were compared to full composition ST266 in a standardized in vitro cell proliferation assay as well as the mouse EAE model of optic neuritis.

## Materials and methods

### Animals/Ethics statement

Female C57BL/6J mice were purchased from the Jackson Laboratory (Bar Harbor, ME, USA). All experiments in this study adhered to the Association for Research in Vision and Ophthalmology Statement for the Use of Animals in Ophthalmic and Vision Research and were compliant with the University of Pennsylvania Institutional Animal Care and Use Committee guidelines and policies. All animal studies and procedures reported in this manuscript were reviewed by the University of Pennsylvania Institutional Animal Care and Use Committee, who specifically approved these studies as the University of Pennsylvania Institutional Animal Care and Use Committee protocol #804701.

### ST266 fractions

Filtrates of ST266 were prepared using Amicon Ultra-15 filters (50 kDa Cat#:UFC905024 and 30 kDa Cat#:UFC903024; Millipore Sigma, St. Louis, MO). The centrifuge (Sorvall Legend RT) was pre-cooled for 4°C and set to 4,000 g. For each filter, depending on the molecular weight cut off, spin time was optimized following manufacturer instructions. Filters were first washed with 15 mL Water for Injection (WFI) to remove potential residual compounds following manufacture. Water was removed, and samples loaded into the filter.

### Schwann cell proliferation assay

For Schwann cell proliferation assay, SW10 Mouse Schwann Cells (Cat# CRL-2766; ATCC, Manassas, VA) were seeded in 96-well tissue culture treated plates and cultured in normal growth media (NGM), containing Dulbecco’s Modified Eagle Medium (DMEM; Cat# 11054–020, Thermo Fisher Scientific, Waltham, MA), GlutaMAX (Cat# 35050061) and 10% FBS (HyClone Cat# SH30071.01, GE Healthcare, Chicago, IL), for a fixed amount of time at 37°C and 5% CO_2_. When acclimated, NGM media was replaced by minimal starvation medium for 24-hours and subsequently replaced by one of the treatment medias: NGM, Iscove’s Modified Dulbecco’s Media (IMDM; growth under minimal conditions), STM100 (proprietary base medium for culturing human placental AMP cells), ST266 or a filtrate fraction of ST266 below the molecular weight cut-off of either 50 kDa or 30 kDa.

After 24-hours, proliferation of Schwan cells was determined using a commercial viable cell counting kit (Cat# 96992, Milipore Sigma, St. Louis, MO) per the manufacturer’s protocol. This colorimetric assay measures the water-soluble formazan dye formed by NADH-mediated reductions in extracellular tetrazolium salt WST-8. ST266 reference control and unconditioned media reference control were also run in each assay. Absorbance was read at 450nm using Synergy 2 plate reader (Bio Tek, Winooski, VT) and either the STM100 or NGM blank media signal was subtracted. For each plate, proliferation was normalized to NGM treatment and data reported as a ratio of sample absorbance to the NGM value. Experiments were repeated three times, each with freshly made ST266 filtrate fractions.

### EAE induction

Chronic experimental autoimmune encephalomyelitis was induced in mice as previously described [9]. Briefly, at age eight weeks, mice were anesthetized with isoflurane and a sub-cutaneous injection was done at two dorsal sites containing a total of 200 μg myelin oligodendrocyte glycoprotein (MOG) peptide (MOG 35–55; Genscript, Piscataway, NJ, USA) emulsified in Complete Freund’s Adjuvant (Difco, Detroit, MI, USA) with 2.5 mg/ml killed Mycobacterium tuberculosis (Difco). Control mice were injected with equivalent volumes of phosphate buffered saline (PBS) accompanied by equal doses of Complete Freund’s Adjuvant and M. tuberculosis. Each animal was also injected intraperitoneally with 200 ng pertussis toxin dissolved in 0.1 ml PBS at the time of initial immunization and again 48 hours later. Disease severity based on ascending paralysis was scored daily using previously described scales as follows: no disease = 0; partial tail paralysis = 0.5; tail paralysis or waddling gait = 1.0; partial tail paralysis and waddling gait = 1.5; tail paralysis and waddling gait = 2.0; partial limb paralysis = 2.5; paralysis of one limb = 3.0; paralysis of one limb and partial paralysis of another = 3.5; paralysis of two limbs = 4.0; moribund state = 4.5; death = 5.0 [9]. Mice were scored and weighed daily for the entire 6 week experiments. While moribund state and death are included in our scoring scale and any mouse reaching a score of 4.5 or losing 20% body weight would be humanely euthanized, the amount of antigen used to induce disease induces mild-moderate disease and is not expected to reach these endpoints. Indeed, no mice were euthanized for humane endpoints and no mice died. The paralytic disease itself is not painful, but for any mice that developed hind limb paralysis moistened food and gel were provided on the floor of their cages to avoid stress of having to reach up to eat or drink.

### ST266 treatment

Aliquots of ST266 prepared as described above for in vitro cell proliferation assays were provided by Noveome Biotherapeutics, Inc., (Pittsburgh, PA, USA) and stored at 4°C. For intranasal dosing, unanesthetized mice were secured by the scruff and 20 μl of the test agent or vehicle control were instilled in the nares once daily, similar to prior studies [4–6].

### Optokinetic response measurement

Optokinetic nystagmus reflex was used to estimate visual performance in mice using the OptoMotry apparatus and software (CerebralMechanics Inc., Medicine Hat, Alberta, CA). As described in prior studies [10], mice were positioned on a platform surrounded on all sides by video monitors displaying 100% contrast sinusoidal black and white bands rotating clockwise or counter clockwise. A trained masked observer graded head movement in the direction of rotation of the bands to detect the threshold spatial frequency (cycles per degree) where an animal fails to track the pattern [11].

### Retinal ganglion cell immunolabeling and quantification

RGCs were quantified by Brn3a immunolabeling using previously described methods [12]. In summary, following euthanasia, eyes were removed and fixed in 4% paraformaldehyde at 4°C overnight. Retinas were dissected, washed with PBS containing 0.5% Triton X-100, and then permeabilized by freezing at -80°C for ten minutes. After thawing, each retina was labeled with rabbit anti-mouse Brn3a antibody (Synaptic Systems #411003, Goettingen, Germany) at 1:4000 dilution in blocking buffer containing PBS with 2% bovine serum albumin and 2% Triton X-100. After overnight incubation at 4°C, retinas were washed four times in PBS and then incubated for one hour at room temperature with an anti-rabbit secondary antibody conjugated to Alexa Fluor 488 (A21206, Thermo Fisher Scientific, Waltham, MA, USA) at 1:4000 dilution in blocking buffer. After washing in PBS x 4, retinas were flat-mounted with four radial relaxing cuts and placed RGC side up on positively charged slides and cover-slipped with vectashield antifade mounting media (Vector Laboratories, Burlingame, CA). Photomicrographs were obtained with an epifluorescent microscope at 20x magnification by an investigator masked to treatment groups. Three representative regions were captured in each quadrant—corresponding to one-sixth, three-sixths, and five-sixths of the retinal radius—for a total of twelve photos per retina. Digital images were re-labeled with random codes for masking and RGCs were semi-automatically counted using established protocols with ImageJ Fiji open source image analysis software [13, 14]. This strategy employs initial automatic detection of RGCs based on size, contrast and shape, to allow for higher throughput and minimal operator bias. To verify good quality analysis, automatically counted images were individually evaluated in a masked manner and counts were manually adjusted in the rare instances this was necessary, for example, if some debris was present on the slide.

### Optic nerve histology

Optic nerves were isolated at the time of sacrifice, fixed in 4% paraformaldehyde, embedded in paraffin, and cut into 5 μm thick longitudinal sections. For assessment of inflammation, sections were stained with H&E and examined by light microscopy. Scores were assigned to each sample by a masked observer per prior studies [15] as follows: no infiltration = 0; mild cellular infiltration of the optic nerve or optic nerve sheath = 1; moderate infiltration = 2; severe infiltration = 3; massive infiltration = 4. While not specifically staining inflammatory cells, prior studies have shown that detection of gross cellularity within the optic nerve on H&E stained sections correlates highly with immunostaining for macrophage markers in EAE optic neuritis [4, 5]. To detect demyelination, sections of the optic nerve were stained with luxol fast blue (LFB) and quantified on a 0–3 point relative scale by a masked investigator as in prior studies [4, 16]: 0 = no demyelination; 1 = scattered foci of demyelination; 2 = prominent foci of demyelination; and 3 = large (confluent) areas of demyelination. The entire length of each optic nerve section was examined.

### Statistics

Evaluation of EAE severity scores and OKR thresholds over time were compared using ANOVA of repeated measures followed by Tukey post-hoc comparisons between each group. RGC counts, final OKR scores, optic nerve inflammation scores, optic nerve demyelination scores, and Schwann cell proliferation levels were compared by one-way ANOVA with Tukey post-hoc comparisons between treatment groups. Where indicated, pairwise comparisons were made using the two tailed Student t-test. For the grouped analysis isolating mice with moderate demyelination phenotype, data from three separate experiments were pooled. In order to avoid potential bias from possibly variable RGC counts in different experiments, mice of each treatment group were compared to untreated controls from their corresponding individual experiment, and this is reported as a ratio, which was then combined between all three experiments in an effort to decease noise. All computations were done using Graph Pad Prism (GraphPad Software, San Diego, CA). P values less than 0.05 were considered significant.

## Results

### ST266 increases Schwann cell proliferation following starvation conditions more potently than either <50 kDa or <30kDa filtrate fractions

**Fig 1:**
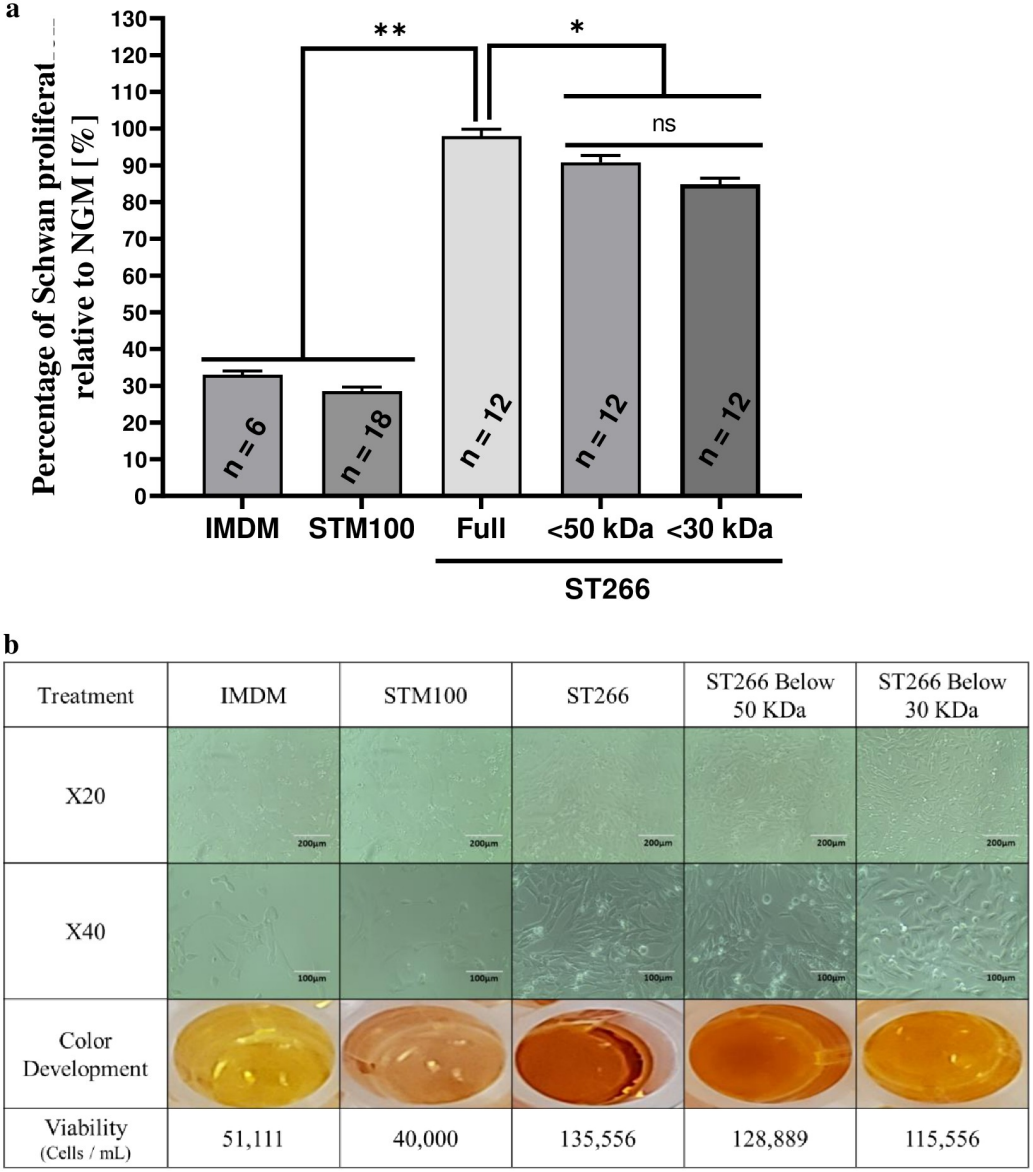
ST266 increases Schwan cell proliferation following 24-hours starvation conditions. Media of Schwan cells cultured in normal growth media (NGM) was replaced by minimal starvation medium for 24-hours and subsequently replaced by either NGM, IMDM, STM100 (base medium), ST266 or filtrate of ST266 below 50- or 30- kDa cutoff. (a) After 24-hours treatment, cell proliferation was determined by extracellular reduction of WST-8, with results shown as a ratio of sample absorbance to the NGM value for each plate. (b) Representative light micrograph images of cultured murine Schwann cells following starvation and a 24-hours treatment demonstrate relative presence of healthy, elongated Schwann cells at 20X (scale bars = 200 μm) and 40X (scale bars = 100 μm) magnification. Images illustrating the development of color from CCK-8 assay in Schwann cell cultures. Corresponding cell viability is listed below each representative set of images. Data shown as mean + SEM from three separate experiments with total of n = 12 per group (**p < 0.0001 and *p < 0.05).

One-way ANOVA with six groups revealed significant differences in Schwann cell proliferation between treatments (F[5,66] = 424.1, p < 0.0001; Fig 1). Tukey’s Post-hoc analysis revealed that Schwann cell proliferation is significantly higher with treatment of ST266 or either of the ST266 filtrate fractions compared to IMDM and STM100 controls (p < 0.0001 for all comparisons). The proliferation effects of the <50 kDa and <30 kDa ST266 fractions were significantly lower than non-fractionated ST266 (p = 0.032 and p < 0.0001, respectively). No significant difference was found between the effect of <50 kDa and <30 kDa fractions (p = 0.11, ns).

### ST266 and <50 kDa fractionated ST266 suppress optic nerve demyelination in mice with mild EAE/optic neuritis

**Fig 2:**
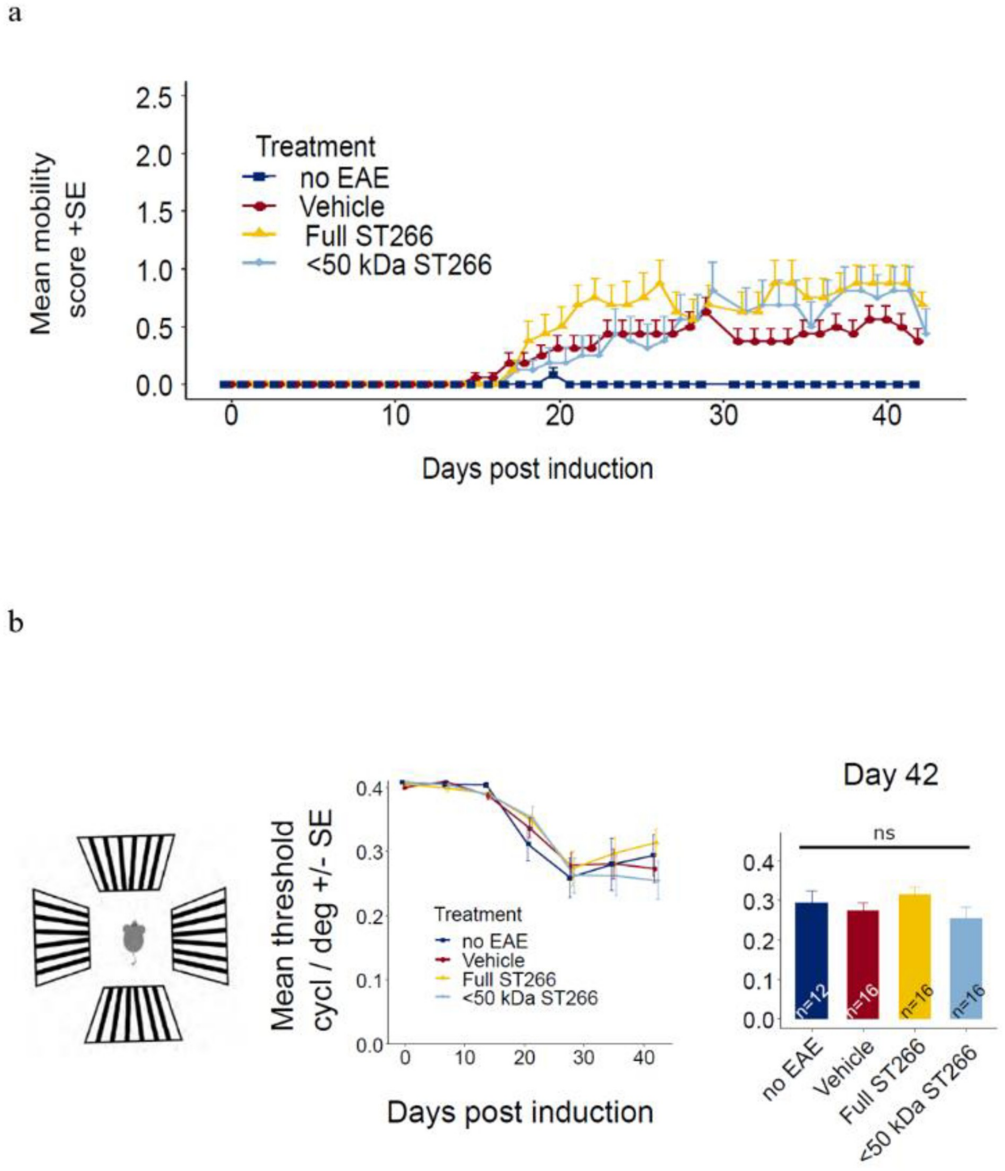
Mild EAE induced by MOG peptide immunization. (a) Mice immunized with MOG peptide to induce EAE were treated daily with intranasal ST266 (n = 8 mice), <50 kDa ST266 (n = 8) or PBS (vehicle) (n = 8) from days 15–42 post-immunization, and control, non-EAE mice (n = 6) were treated with PBS. Mice were scored daily based on clinical signs of ascending paralysis. Mild EAE disease developed in all immunized groups with no difference in EAE scores between treatment groups (one-way repeated measures ANOVA p>0.05). (b) To estimate visual function, OKR testing was performed. Measurements were taken at baseline and then weekly for 6 weeks. In this cohort of mice with mild EAE disease, no significant vision loss developed compared with control mice, and no difference was detected in mean scores between treatment groups over time (one-way repeated measures ANOVA p>0.05) or at the final measurement on day 42 (one way ANOVA p >0.05). Data shown as mean +SEM.

**Fig 3:**
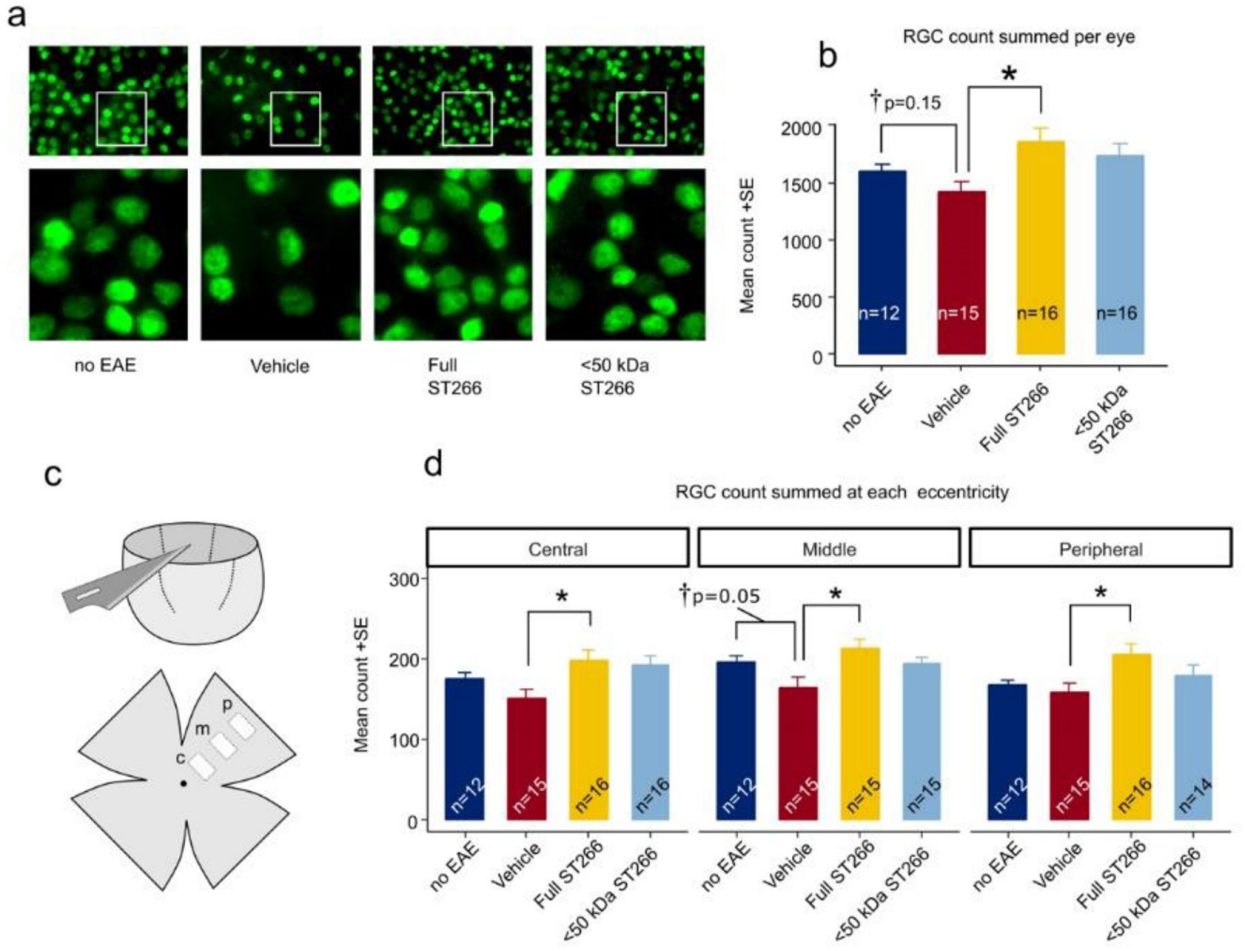
RGC neuroprotection by ST266 in mild EAE optic neuritis. (a) Representative photos of Brn3a immunolabeled RGCs in flat mounted retinas isolated 42 days post-immunization from the same cohorts of mice shown in Fig 2. Lower magnification images (top; original magnification X 10) demonstrate the density of RGCs. White boxes indicate areas shown at higher magnification (bottom) demonstrating the ability to identify individual Brn3a positive cells. (b) Total number of RGCs across 12 standardized fields for each eye show a trend toward loss of RGCs in eyes from PBS-treated EAE mice (n = 16 eyes) compared with eyes from control mice (n = 12 eyes) (student t-test †p = 0.155). Eyes from EAE mice treated with ST266 (n = 16) showed greater RGC counts than vehicle-treated EAE animals (ANOVA *p<0.05), whereas the trend toward increased RGC numbers in eyes from <50 kDa ST266-treated EAE mice (n = 16) compared with PBS-treated EAE mice was not significant. (c) Diagram shows the eccentricity of standardized photos of RGCs, including a central (c), mid-peripheral (m) and peripheral (p) photo which were obtained in each retinal quadrant. (d) Average RGC numbers present in each retinal region show that eyes from mice treated with ST266 have greater numbers of surviving RGCs than eyes from PBS-treated EAE mice (one-way ANOVA *p<0.05) in all three retinal regions. The mid-peripheral region showed the greatest difference in RGC numbers between eyes from control mice and eyes from PBS-treated EAE mice, although this was not significant by ANOVA but was significant in direct comparison between these two groups (student t-test †p = 0.05). Data shown as mean +SEM.

**Fig 4:**
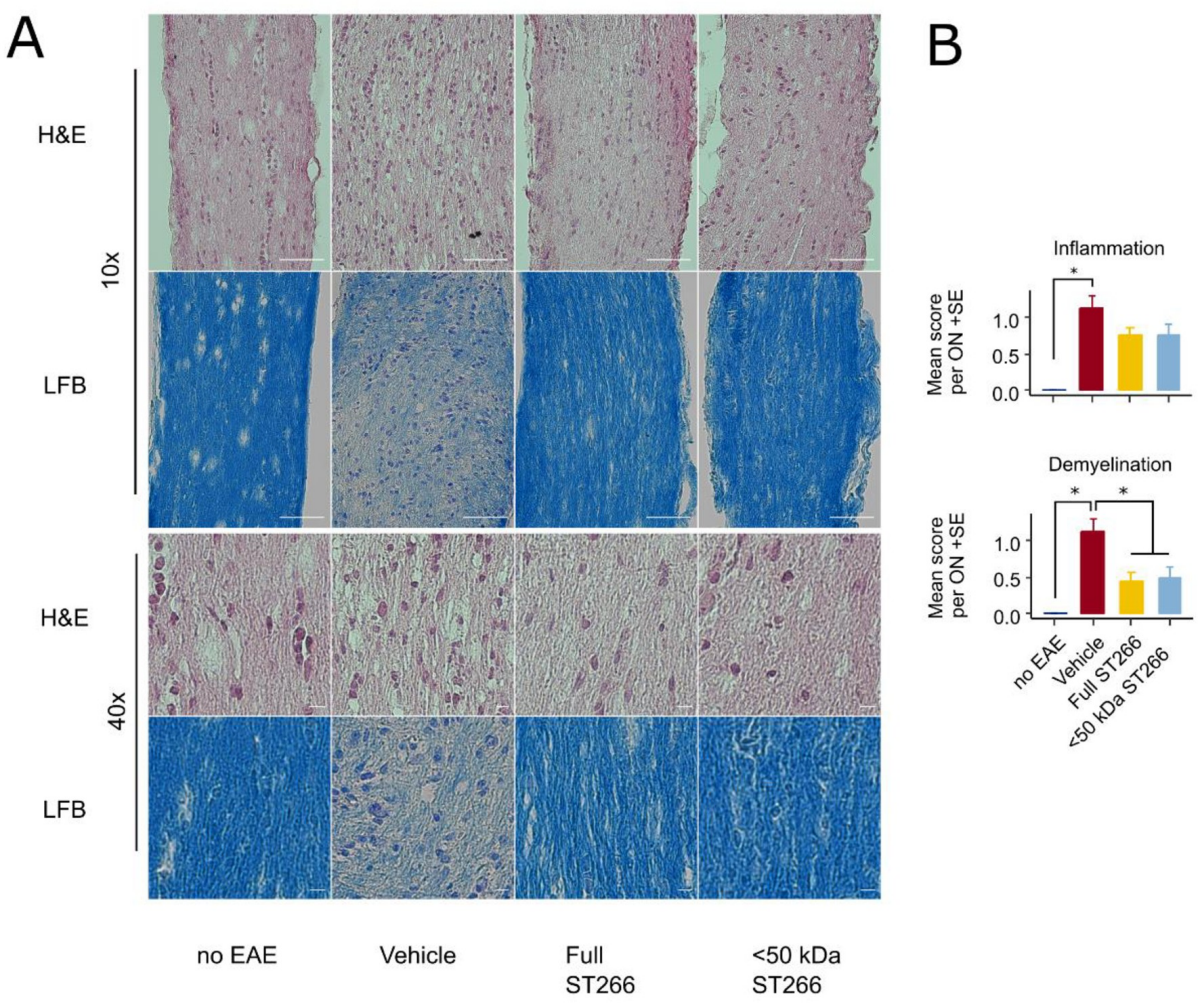
Optic nerve demyelination is significantly reduced by both ST266 and <50 kDa ST266 in mild EAE optic neuritis. (a) Representative photos of optic nerve sections are shown from the same cohorts of mice shown in Fig 2, prepared following sacrifice on day 42 post-immunization. H&E staining shows the relative cellularity within the optic nerves, and LFB staining shows the relative level of myelination. Scale bar = 50 microns in lower magnification images (top two rows) and scale bar = 5 microns in higher magnification images (bottom two rows). (b) Masked scoring demonstrates increased inflammatory cell infiltration present in nerves from PBS-treated EAE mice (n = 16 optic nerves) as compared with nerves from control, non-EAE mice (n = 12) (*p<0.05). Daily intranasal ST266 (n = 16) or <50 kDa ST266 (n = 16) treatment both show a strong trend towards reducing optic nerve inflammation that is not statistically significant as compared with PBS-treated EAE mice. Masked scoring of LFB staining demonstrates increased levels of myelin loss present in nerves from PBS-treated EAE mice (n = 16 optic nerves) as compared with nerves from control, non-EAE mice (n = 12) (*p<0.05). Daily intranasal ST266 (n = 16) or <50 kDa ST266 (n = 16) treatment both show significant reduction in optic nerve demyelination as compared with PBS-treated EAE mice (*p<0.05). Comparisons analyzed by one-way ANOVA with Tukey post-hoc testing. Data shown as mean +SEM.

EAE mice (n = 8/treatment group) and control (non-EAE) mice (n = 6) were monitored daily for development of ascending paralysis and weekly for OKR responses, prior to sacrifice at day 42 post-immunization. EAE induction produced only mild EAE disease which was not altered by daily intranasal treatment with ST266 or <50 kDa ST266 (Fig 2a), and failed to induce a significant decrease in OKR responses (Fig 2b). However, even in this cohort of mice with only mild EAE disease, an overall trend toward loss of RGCs in EAE mice was observed, and analysis of RGC survival by retinal region (central, mid-peripheral, and peripheral) showed a small but significant loss of RGCs induced by EAE in the mid-periphery (Fig 3). This regional RGC loss was significantly prevented by daily treatment with ST266, whereas <50 kDa ST266 treatment only led to a non-significant trend towards increased RGC survival as compared to PBS-treated EAE mice (Fig 3). EAE mice developed significant optic nerve inflammation and demyelination, and treatment with both ST266 and <50 kDa ST266 significantly reduced the level of demyelination compared with PBS-treated EAE mice. Both treatments prevented significant optic nerve inflammation from developing as compared to control, non-EAE mice, but showed only a trend towards reducing inflammation compared to PBS-treated EAE mice (Fig 4).

### ST266 attenuates decreases in OKR responses and loss of RGCs in EAE mice whereas effects of <50 kDa ST266 are not significant

To assess effects of ST266 and <50 kDa ST266 in mice with moderately higher levels of EAE disease, the experiment above was repeated two additional times, and all EAE mice that developed mild to moderate clinical EAE disease were used to assess optic neuritis outcomes. All mice that developed a cumulative EAE paralysis score between 0.5–60 (EAE score summed over entire 42 days of experiment) were included in this study. Mice that failed to develop any clinical signs of EAE paralysis (EAE score of 0) and mice who developed severe EAE disease defined as either a cumulative EAE score >60, or a single day EAE score of 4 (both hind limbs paralyzed) or higher, were excluded. In all, 44 eyes of 22 control (non-EAE mice) were examined and compared to 23 eyes from EAE mice treated with PBS, 26 eyes from EAE mice treated with ST266, and 24 eyes from mice treated with <50 kDa ST266.

**Fig 5:**
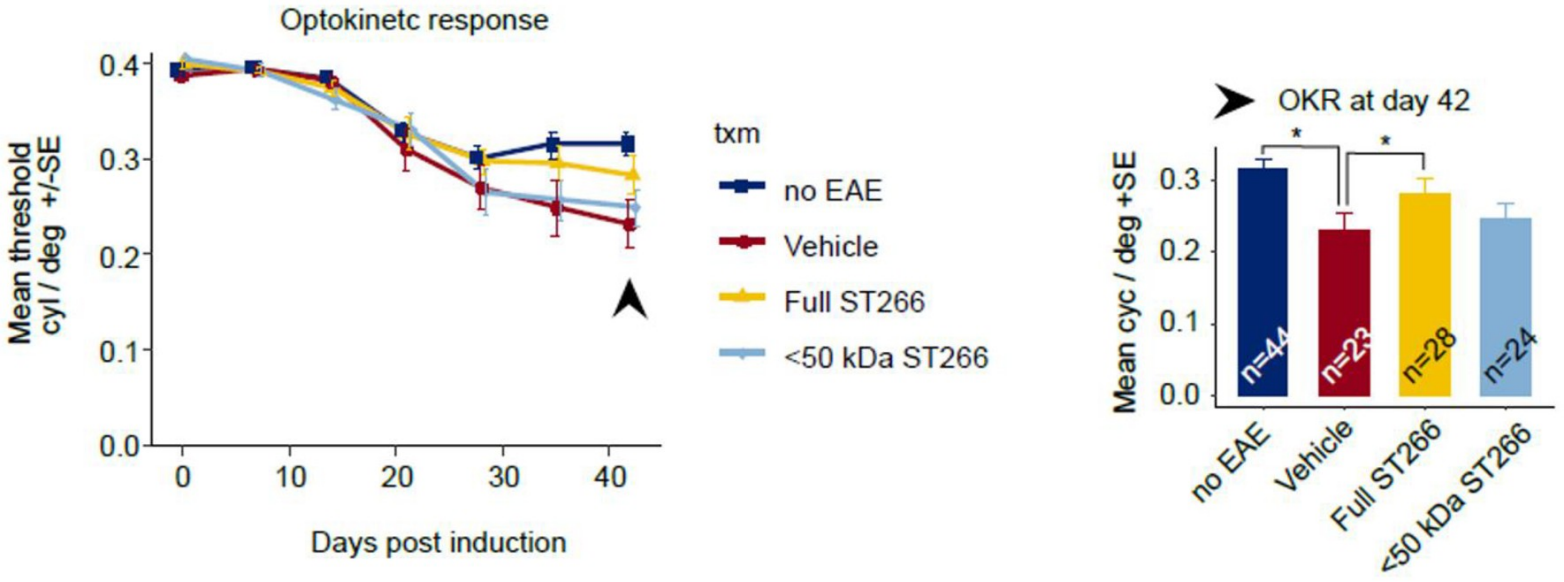
ST266 preserves visual responses in mice with mild-moderate EAE optic neuritis. Mice immunized with MOG peptide to induce EAE were treated daily with intranasal ST266 (n = 24 mice), <50 kDa ST266 (n = 24) or PBS (n = 24) from days 15–42 post-immunization, and control, non-EAE mice (n = 22) were treated with PBS across three identical experiments. Eyes from mice that developed at least minimal EAE paralysis (cumulative EAE score greater than 0 over 42 days post-immunization) but without severe disease (cumulative EAE score not more than 60) were included for further analysis of visual function. OKR scores measured weekly across 42 days showed a non-significant trend towards improved responses in eyes from EAE mice treated with ST266 as compared with eyes from PBS-treated EAE mice when compared by ANOVA of repeated measures. At day 42 post-immunization, a significant decrease in OKR responses in eyes from PBS-treated EAE mice (n = 23 eyes) was observed as compared to eyes from control, non-EAE mice (n = 44) (*p<0.05), and this decrease was significantly improved in eyes from ST266-treated EAE mice (n = 28) (*p>0.05), while a trend towards improvement in eyes of <50 kDa ST266-treated mice (n = 24) did not reach statistical significance. Data shown as mean +SEM.

**Fig 6:**
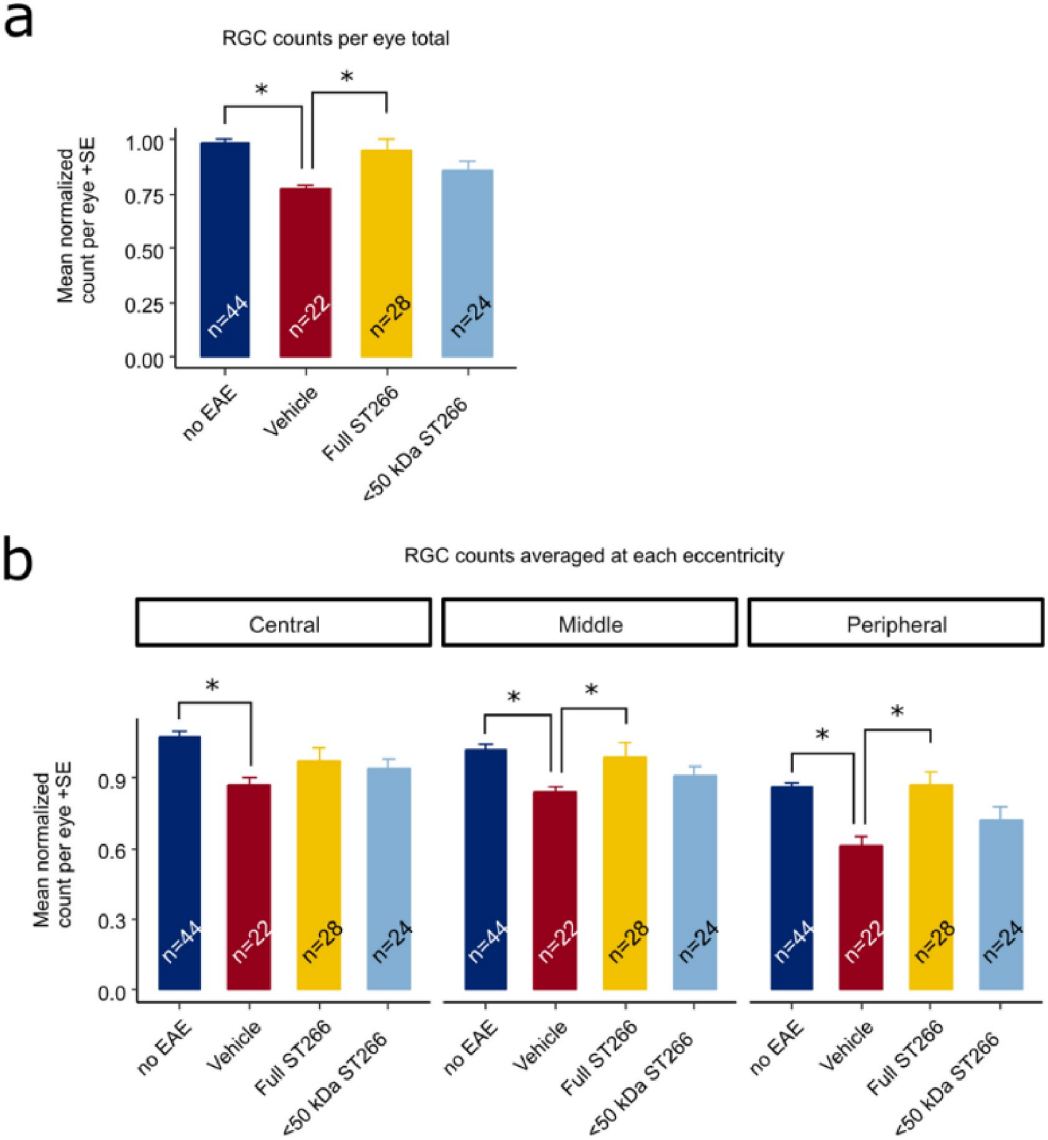
ST266 attenuates RGC loss in mice with mild-moderate EAE optic neuritis. Retinas were isolated from the eyes of control mice and EAE mice with mild-moderate EAE disease and immunolabeled with Brn3a. Data are from the same eyes shown in Fig 5, with the exception of one eye from the vehicle (PBS)-treated EAE mouse cohort in which the retina was damaged during dissection and therefore could not be quantified. (a) The average normalized RGC count across the entire retina shows a decrease in RGC numbers in vehicle-treated EAE mouse eyes (n = 22 eyes) compared to eyes from non-EAE control mice (n = 44 eyes) (*p<0.05). RGC numbers were significantly higher in eyes from ST266-treated EAE mice (n = 28) as compared with PBS-treated EAE mice (*p<0.05), whereas treatment with <50 kDa ST266 (n = 24 eyes) led to a non-significant trend towards increased RGCs. (b) RGC numbers in central, mid-peripheral and peripheral regions of the retina showed a significant decrease in all regions in eyes of PBS-treated EAE mice as compared with control mouse eyes, and treatment with daily intranasal ST266 improved RGC survival in both the mid-peripheral and peripheral retinal regions (*p<0.05). Data shown as mean +SEM.

Over the course of 42 days, progressive decline in OKR responses were observed in EAE mice, with a trend toward improved OKR in EAE mice treated with ST266 and <50 kDa ST266 that was not significant by ANOVA of repeated measures. On Day 42 post-immunization, OKR responses were significantly decreased in PBS-treated EAE mice as compared with control, non-EAE mice, and daily ST266 treatment significantly improved OKR responses whereas <50 kDa ST266 treatment led to a non-significant trend in improved OKR responses (Fig 5). ST266 also significantly attenuated EAE-induced loss of RGCs (Fig 6a), and specifically improved RGC survival in the mid-periphery and peripheral retina (Fig 6b). Treatment with <50 kDa ST266 lead to a non-significant trend toward increased RGC survival (Fig 6).

## Discussion

Results demonstrating the ability of ST266 to reduce RGC loss and preserve functional visual responses measured by OKR in EAE mice are consistent with prior studies showing similar neuroprotective effects of intranasal ST266 in EAE optic neuritis [4, 5]. Removal of the highest molecular weight components of ST266, >50 kDa, led to diminished neuroprotective effects in EAE optic neuritis, as this induced a trend toward increased RGC survival and preserved OKR responses, but that trend was not significant. ST266 did however maintain a significant ability to reduce levels of demyelination in mice with mild EAE disease even after removal of >50 kDa proteins. In vitro, fractionated <50 kDa and <30 kDa ST266 each showed a significant ability to stimulate Schwann cell proliferation, but stimulated significantly less proliferation than non-fractionated ST266. Together, these studies demonstrate that the lower molecular weight fraction of ST266 retains some effects mediated by complete ST266, both in vitro and in an in vivo disease model, but not all effects, suggesting that the full complement of biomolecules present in ST266 may provide the optimal combination of factors to promote important biologic effects.

Current results build on previous work showing decreased myelin loss and protection against RGC death in optic neuritis and traumatic optic neuropathy [4, 6] as well as benefits in other models of inflammation and wound healing [17–20]. The data suggests that the mechanism of action is due to a combination of factors and not from a single molecule. In a clinical study of UV-induced skin damage, the DNA repair enzymes such as Xeroderma pigmentosum, complementation group A (XPA) and cyclobutane pyrimidine dimer (CPD) were potentially implicated in ST266 treatment [18]. In optic neuritis, the SIRT1 pathway was shown to be activated in the setting of ST266 treatment and could be acting by increasing mitochondrial biogenesis via PGC1α [4]. While these pathways could be important, it is not well understood which molecules in ST266 are driving upregulation of these pathways, and it is likely that multiple factors play a role.

Known components of ST266 itself reveal hundreds of cytokines and growth factors which have been implicated in anti-inflammatory and neuro protective pathways. Of these, the majority are less than 50 kDa in size, including platelet derived growth factor BB—which has been shown to protect against RGC loss along with Igf1 via Akt [21]—as well as angiogenin, macrophage inhibitory cytokine-1 (MIP1), macrophage inhibitory cytokine-1 (MIP1), dipeptidyl peptidase-IV(DPP4), soluble tumor necrosis factor receptor 1, soluble tumor necrosis factor–related apoptosis-inducing ligand receptor-3, Axl, tissue inhibitors of metalloproteinases (TIMP1 and TIMP2) and the lipid resolvin D1 [3]. Of note, this panel of possible targets is not complete, and future comprehensive proteomic studies of ST266 will be useful.

As discussed in prior reports, the effect of ST266 likely relies on a multitude of simultaneous paracrine signals—as each component is present in physiologic or sub-physiologic concentrations [3]. In contrast, prior studies of neuroprotection in the eye which sought to target specific pathways have not historically proved clinically successful. Small molecules such as brimonidine and memantine as well as single growth factors such as ciliary neurotropic factor (CNTF) failed to demonstrate clinical utility in human retinal and optic nerve disease [22]. Glucocorticoids, which likely have pleotropic targets, have been shown to decrease inflammation in EAE mice, but do not protect against RGC loss when they are initiated after symptom onset [23]. Our approach of using the secreted products of a defined cell population is similar to work where whole platelet lysate was advantageous in wound healing [24]. Mesenchymal stem cell treatments [25], as well as exercise [26] are hypothesized to employ a similar mechanism of trophic support via a host of growth factors and cytokines to create a desired cellular milieu and have shown promising results in neurodegenerative models. Our strategy builds on this model starting with a reproducible mixture of growth factors derived from a biological source and now seeks to optimize this agent for potential therapeutic use.

Compared to other pleiotropic strategies for neuroprotection, the non-invasive, intranasal dosing of ST266 offers pharmacokinetic advantages which highlight the importance for optimization of this route. Local diffusion along the olfactory nerves or lymphatic channels is suspected to play a role in the accumulation of ST266 to the vitreous and optic nerve based on radiotracer studies showing optic nerve accumulation as soon as 30 minutes after nasal dosing in the rat [4]. Furthermore, prior studies showed isolated benefits of ST266 to the optic nerve and not the spinal cord, suggesting that effects are primarily local and not systemic [4]. Alternatively, the absorbed dose may not have been sufficient to attenuate EAE paralysis. CNS drug delivery intranasally has been demonstrated even with large growth factors [27]. We have shown here that removing proteins >50 kDa may not preserve all effects of ST266, and future strategies for drug delivery should likely focus on the delivery of the full complement of ST266 biomolecules.

In this study, the initial optic neuritis experiment (Figs 2–4) showed the degree of ST266 treatment effect was somewhat less than prior reports [4, 5] which is likely due to the mild severity of EAE disease induced. EAE is an inherently variable disease and in this case produced only limited optic nerve pathology, which provides important data showing that even with mild disease, ST266 can still provide benefits. It has been shown that optic neuritis can occur unilaterally (>40%), bilaterally (>40%) or not at all (15%) in EAE mice [28]. Thus, for this and subsequent experiments, each eye was analyzed as an independent data point similar to prior studies [4, 5, 16, 23]. To further assess ST266 treatment effects in mice with more typical mild-moderate disease severity, we analyzed data from cohorts of mice with mobility scores greater than zero (at least some mobility deficit) and less than 60 (cumulative EAE scores over 42 days). As intranasally-delivered ST266 treatment has previously been shown not to affect EAE spinal cord disease, EAE paralysis scores were used as a way to independently adjust for variability in disease severity in mice. Using this approach, we demonstrated that ST266 effects are similar to prior reports in terms of RGC survival and functional vision preservation as measured by OKR [4, 5]. Lower molecular weight ST266 (<50 kDa) showed strong trends but not a significant benefit compared with sham treated animals and thus may retain some neuroprotective properties but appears to be less useful than full complement ST266.

Overall, results here further support intranasal ST266 as a candidate neuroprotective therapy for optic neuritis, and suggest that development of strategies to deliver the full complement of biomolecules present in ST266 will be important for future clinical applications.
